# Pitfalls in Modeling Asymptomatic COVID-19 Infection

**DOI:** 10.3389/fpubh.2021.593176

**Published:** 2021-04-12

**Authors:** Shujuan Yang, Shaoqing Dai, Yuling Huang, Peng Jia

**Affiliations:** ^1^West China School of Public Health and West China Fourth Hospital, Sichuan University, Chengdu, China; ^2^International Institute of Spatial Lifecourse Epidemiology (ISLE), Hong Kong, China; ^3^Sichuan Center for Disease Control and Prevention, Chengdu, China; ^4^Department of Land Surveying and Geo-Informatics, The Hong Kong Polytechnic University, Hong Kong, China

**Keywords:** COVID-19, epidemiology, asymptomatic infection, disease modeling, disease transmission

As the supply of nucleic acid detection kits for coronavirus disease 2019 (COVID-19) is gradually approaching the demand for testing those with moderate or severe symptoms, even those with mild symptoms of the common cold in some countries, research interests in scientific communities have started to shift to those unidentified COVID-19 infections presenting mild or even no symptoms (i.e., asymptomatic COVID-19 infections). The observed rates of asymptomatic COVID-19 infections have varied from 17% in a family of six to 67% in a family of three (1, 2). Although such observational studies are subject to the limited sample size and selection bias, the emerging studies estimating the rates of unidentified COVID-19 infections may be subject to more severe biases or even errors, if we do not have a deep understanding of unidentified COVID-19 infections in the context of efforts of epidemic control and prevention on the ground.

For example, a recent study simulating the dynamics of coronavirus disease 2019 (COVID-19) infection during 10–23 January 2020 revealed “a very high rate of undocumented infections: 86%” (3). However, such an estimate should be treated with caution because several flaws in fundamental definitions and assumptions in that study can significantly affect the accuracy and implications of the results. A most basic flaw is that they “divided infections into two classes: (i) documented infected individuals with symptoms severe enough to be confirmed, i.e., observed infections; and (ii) undocumented infected individuals.” In this seemingly reasonable procedure, authors mixed up three concepts, i.e., undocumented, unconfirmed, and unnoticed, which has fundamentally undermined the accuracy of that study and largely accounted for why that estimate has not been validated or even approached by any observational study so far. Such flawed definitions and assumptions would also undermine the quality of many more, if not all, forthcoming scientific studies in that direction and, more importantly, mislead general readers in understanding what has happened at the beginning of the COVID-19 pandemic in other provinces than Hubei Province of China. Therefore, they deserve some factual explanations.

First, the rate of “undocumented” infections is not an estimatable concept. It would have been more appropriate to estimate the rate of “unnoticed” infections (i.e., asymptomatic infections) or “unconfirmed” infections (i.e., untested infections) (4). In particular, authors have clearly explained the first class as “observed” infections, then the second class was actually, also naturally, “unobserved” infections, which should be more rigorously defined as “unnoticed” infections or, at the very least, less rigorously defined as “unnoticed” or “unconfirmed” infections. In any case, conceptually, scientifically, or literally, the second class should have been correctly defined. Failure to do so has further led to other problematic statements and assumptions in the following.

Second, observed infections were equal to “documented infected individuals with symptoms severe enough to be confirmed,” which was problematic due to lack of a clear definition of “symptoms severe enough.” Such vagueness also existed in another statement that “these undocumented infections often experience mild or limited symptoms and hence go unrecognized,” where “mild” and “limited” symptoms were not clearly defined. A reasonable guess is that authors did not have sufficient knowledge of COVID-19 symptoms at the time of conducting this study, according to the entire lack of early epidemiological studies including clinical characteristics of COVID-19 patients in the reference list of that article (5–7). Defining the epidemiology of COVID-19 on the basis of a large enough sample of infected cases (8) or their spatiobehavioral characteristics (i.e., individuals' close contact with infectors for a certain amount of time) (9), and thereby understanding the characteristics of COVID-19 (i.e., elucidating what the full spectrum of disease severity is, how transmissible the virus is, who the infectors are), is a critical step prior to any reliable scientific study examining the transmission and impact of COVID-19. This is also why very few (reliable) studies, if not entirely lacking, in this direction have been conducted outside China before March 2020. Therefore, simulating the dynamics of symptomatic and asymptomatic COVID-19 infections without considering the definitions of COVID-19 symptoms has invalidated that study to some extent.

Third, the statement that “these undocumented infections often experience mild or limited symptoms and hence go unrecognized” is not true in all provinces but Hubei, where most, if not all, infections presenting mild symptoms in other provinces have visited their nearby fever clinics and been tested at the first moment possible (10–12); thus, they could not have gone unrecognized. Only some of those experiencing mild or limited symptoms prior to 23 January in Hubei Province, especially in Wuhan, may not be timely tested for COVID-19, due to insufficient capacity of conducting COVID-19 nucleic acid testing at that time; even so, most infections presenting any symptom have visited their local fever clinics and been triaged for at-home isolation or in-hospital isolation and treatment (11). Therefore, “the transmission rate due to undocumented individuals” in that study, in the simplest assumption, should be different for “unconfirmed” and “unnoticed” infections rather than a constant value for both groups of infections. This mistake could simply occur because authors failed to differentiate “unconfirmed” from “unnoticed” infections among “undocumented.” The transmission rate among “unconfirmed” infections, if not zero, should be much lower than that of “unnoticed” infections, which invalidated authors' another statement that “those often experiencing mild, limited or no symptoms, depending on their contagiousness and numbers, can expose a far greater portion of the population to virus than would otherwise occur”. In addition, there have been many intrapersonal and interpersonal variations in the contagiousness over time, which could not be simply hypothesized (13). Also, the number of infections from those variable or unknown contagiousness depends heavily on the physical interaction between people, which could not be realized without the support and integration of individual-level movement trajectory (14, 15).

Fourth, the statement that “these undocumented infections often experience no symptoms and hence go unrecognized” is not aligned with epidemic control and prevention efforts on the ground, and also could not be validated until blanket testing. Since the beginning of the COVID-19 outbreak, the local Centers for Disease Control and Prevention (CDC) in all Chinese provinces except Hubei have been helping infected people experiencing any symptom recall all their potential contacts, who have then been tested while experiencing no symptoms (16). Only a very small number of asymptomatic infections were detected in that way, which added little to the infected population; for example, the infection rates of close contacts of confirmed cases and asymptomatic infections were 6.30% (126 out of 2,001) and 4.11% (6/146), respectively (17). Despite the lack of an overall picture of the number and distribution of asymptomatic infections at the early stage of the epidemic, what had been done at that time for identifying asymptomatic infections in China has been considered acceptable (18).

Last but not least, none of observational studies published so far have validated or even been close to the “undocumented infection rate of 86%” estimated based on those flawed definitions and assumptions. The national health commission of China published that, as of 31 March, 2020, the total number of asymptomatic COVID-19 infections was 1,541 including 205 imported cases (19). A recent observational study revealed that asymptomatic infections contributed to only <5% of the total infected population, and their contagiousness was only 1/6-1/3 of the contagiousness of confirmed infections in Ningbo, Zhejiang Province of China (17). Although the estimates may vary across Chinese cities, the Chinese CDC has confirmed that the result is in the right ballpark. After all, the observed rate of asymptomatic COVID-19 infections was just 30.8% among 565 Japanese people evacuated from Wuhan by 6 February 2020 (20).

Given that blanket testing is currently not possible in most if not all countries, especially during epidemics when healthcare resources are relatively lacking, reporting unvalidated estimates of the rate of unnoticed infections may not be the optimal way in which both policy-makers and citizens should be informed of the severity of epidemics (21). Unvalidated estimates without consideration of epidemic control and prevention efforts on the ground are against the international recognition by the World Health Organization and public health scientists all over the world who have closely followed the emergence of the COVID-19 (18, 22). They could have been exploited by media to harm the scientists, public health professionals, and medical professionals who have “worked diligently and effectively to rapidly identify the pathogen behind this outbreak, put in place significant measures to reduce its impact, and share their results transparently with the global health community” (22). We suggest the involvement of local CDCs in epidemic studies for more unbiased descriptions of the facts and/or more factual assumptions. Science is important, but public health is much more complex than science. After all, an epidemic or pandemic is a public health emergency event happening on the ground, being closely observed and intervened by local CDCs, rather than a mathematical phenomenon (23). Although estimating the magnitude of unnoticed COVID-19 infections, if possible, may not be a bad idea, such estimates should not be exaggerated without solid evidence, especially when we are still in the pandemic. All science requires factual assumptions and validation. Studies lacking both components should be treated with caution.

## Author Contributions

SY and PJ conceived the idea. All authors contributed to the writing and revision of the draft, and approved it for publication.

## Conflict of Interest

The authors declare that the research was conducted in the absence of any commercial or financial relationships that could be construed as a potential conflict of interest.

## References

[1] Bai Y Yao L Wei T Tian F Jin DY Chen L. Presumed asymptomatic carrier transmission of COVID-19. JAMA. (2020) 323:1406–7. 10.1001/jama.2020.2565 32083643 PMC7042844

[2] Pan X Chen D Xia Y Wu X Li TX. Asymptomatic cases in a family cluster with SARS-CoV-2 infection. Lancet Infect Dis. (2020) 20:410–1. 10.1016/S1473-3099(20)30114-6 32087116 PMC7158985

[3] Li R Pei S Chen B Song Y Zhang T Yang W. Substantial undocumented infection facilitates the rapid dissemination of novel coronavirus (SARS-CoV2). Science. (2020) 368:489–93. 10.1101/2020.02.14.20023127 32179701 PMC7164387

[4] Day M. Covid-19: four fifths of cases are asymptomatic, China figures indicate. BMJ. (2020) 369:m1375. 10.1136/bmj.m1375 32241884

[5] Li Q Guan X Wu P Wang X Zhou L Tong Y. Early transmission dynamics in Wuhan, China, of novel coronavirus-infected pneumonia. N Engl J Med. (2020) 382:1199–207. 10.1056/NEJMoa2001316 31995857 PMC7121484

[6] Zhou F Yu T Du R Fan G Liu Y Liu Z. Clinical course and risk factors for mortality of adult inpatients with COVID-19 in Wuhan, China: a retrospective cohort study. Lancet. (2020) 395:1054–62. 10.1016/S0140-6736(20)30566-3 32171076 PMC7270627

[7] Chen H Guo J Wang C Luo F Yu X Zhang W. Clinical characteristics and intrauterine vertical transmission potential of COVID-19 infection in nine pregnant women: a retrospective review of medical records. Lancet. (2020) 395:809–15. 10.1016/S0140-6736(20)30360-3 32151335 PMC7159281

[8] Lipsitch M Swerdlow DL Finelli L. Defining the epidemiology of Covid-19 - studies needed. N Engl J Med. (2020) 382:1194–6. 10.1056/NEJMp2002125 32074416

[9] Yang S Yu C Jia P. Spatiobehavioral characteristics - defining the epidemiology of new contagious diseases at the earliest moment possible. Trends Parasitol. (2021) 37:179–81. 10.1016/j.pt.2020.12.004 33487571 PMC7826086

[10] Zhu W Xie K Lu H Xu L Zhou S Fang S. Initial clinical features of suspected coronavirus disease 2019 in two emergency departments outside of Hubei, China. J Med Virol. (2020) 92:1525–32. 10.1002/jmv.25763 32167181 PMC7228360

[11] Zhang J Zhou L Yang Y Peng W Wang W Chen X. Therapeutic and triage strategies for 2019 novel coronavirus disease in fever clinics. Lancet Respir Med. (2020) 8:e11–2. 10.1016/S2213-2600(20)30071-0 32061335 PMC7159020

[12] Cao Y Li Q Chen J Guo X Miao C Yang H. Hospital emergency management plan during the COVID-19 epidemic. Acad Emerg Med. (2020) 27:309–11. 10.1111/acem.13951 32124506 PMC7159322

[13] He X Lau EH Wu P Deng X Wang J Hao X. Temporal dynamics in viral shedding and transmissibility of COVID-19. Nat Med. (2020) 26:672–5. 10.1038/s41591-020-0869-5 32296168

[14] Jia P. Understanding the epidemic course in order to improve epidemic forecasting. GeoHealth. (2020) 4:e2020GH000303. 10.1029/2020GH000303 33024909 PMC7532285

[15] Jia P Yang S. Time to spatialize epidemiology in China. Lancet Global Health. (2020) 8:e764–5. 10.1016/S2214-109X(20)30120-0 32446343 PMC7241977

[16] Chen S Yang J Yang W Wang C Barnighausen T. COVID-19 control in China during mass population movements at New Year. Lancet. (2020) 395:764–6. 10.1016/S0140-6736(20)30421-9 32105609 PMC7159085

[17] Chen Y Wang A Yi B Ding K Wang H Wang J. The epidemiological characteristics of infection in close contacts of COVID-19 in Ningbo city. Chin J Epidemiol. (2020) 41:668–72. 10.3760/cma.j.cn112338-20200304-00251 32447904

[18] The Lancet Emerging understandings of 2019-nCoV. Lancet. (2020) 395:311. 10.1016/S0140-6736(20)30186-0 31986259 PMC7134625

[19] National Health Commission of China. Control and Prevention of Asymptomatic COVID-19 Infections (2020). Available online at: http://www.nhc.gov.cn/jkj/s3578/202003/718c79c96f3e46409dd49303d41a00ef.shtml

[20] Nishiura H Kobayashi T Suzuki A Jung SM Hayashi K Kinoshita R. Estimation of the asymptomatic ratio of novel coronavirus infections (COVID-19). Int J Infect Dis. (2020) 94:154–5. 10.1016/j.ijid.2020.03.020 32179137 PMC7270890

[21] Song JY Yun JG Noh JY Cheong HJ Kim WJ. Covid-19 in South Korea - challenges of subclinical manifestations. N Engl J Med. (2020) 382:1858–9. 10.1056/NEJMc2001801 32251568 PMC7154984

[22] Calisher C Carroll D Colwell R Corley RB Daszak P Drosten C. Statement in support of the scientists, public health professionals, and medical professionals of China combatting COVID-19. Lancet. (2020) 395:e42–3. 10.1016/S0140-6736(20)30418-9 32087122 PMC7159294

[23] Saltelli A Bammer G Bruno I Charters E Di Fiore M Didier E. van der Sluijs, and Vineis P. Five ways to ensure that models serve society: a manifesto. Nature. (2020) 582:482–4. 10.1038/d41586-020-01812-9 32581374

